# Liquid biopsies to occult brain metastasis

**DOI:** 10.1186/s12943-022-01577-x

**Published:** 2022-05-10

**Authors:** Asad Ur Rehman, Parvez Khan, Shailendra Kumar Maurya, Jawed A. Siddiqui, Juan A. Santamaria-Barria, Surinder K. Batra, Mohd Wasim Nasser

**Affiliations:** 1 Department of Biochemistry and Molecular Biology, University of Nebraska Medical Center, Omaha, NE 68108 USA; 2 Fred and Pamela Buffett Cancer Center, University of Nebraska Medical Center, Omaha, NE 68108 USA; 3 Department of Surgery, University of Nebraska Medical Center, Omaha, NE 68108 USA; 4 Eppley Institute for Research in Cancer and Allied Diseases, University of Nebraska Medical Center, Omaha, NE-68198 USA

**Keywords:** CTCs, Cell-free DNA, microRNA, Brain microenvironment, Cancer diagnostics, Exosomes

## Abstract

Brain metastasis (BrM) is a major problem associated with cancer-related mortality, and currently, no specific biomarkers are available in clinical settings for early detection. Liquid biopsy is widely accepted as a non-invasive method for diagnosing cancer and other diseases. We have reviewed the evidence that shows how the molecular alterations are involved in BrM, majorly from breast cancer (BC), lung cancer (LC), and melanoma, with an inception in how they can be employed for biomarker development. We discussed genetic and epigenetic changes that influence cancer cells to breach the blood-brain barrier (BBB) and help to establish metastatic lesions in the uniquely distinct brain microenvironment. Keeping abreast with the recent breakthroughs in the context of various biomolecules detections and identifications, the circulating tumor cells (CTC), cell-free nucleotides, non-coding RNAs, secretory proteins, and metabolites can be pursued in human body fluids such as blood, serum, cerebrospinal fluid (CSF), and urine to obtain potential candidates for biomarker development. The liquid biopsy-based biomarkers can overlay with current imaging techniques to amplify the signal viable for improving the early detection and treatments of occult BrM.

## Introduction

Tissue biopsy is considered one of the best methods for accurate cyto−/histologic diagnosis, disease grading, and revealing novel targets for personalized cancer treatments [1, 2]. However, as our understanding of cancer over the last decade has unfurled, interpretation of tissue biopsies have proved to be increasingly challenging, not just confined to tissue acquisition, which is more salient when tumors are not accessible, but the major obstacle is the cancer evolution that further adds to tumor heterogeneity, which is why tissue specimen excised never portray a complete picture [3–7]. Interestingly, Kwak et al. reported that treatment on esophagogastric cancer patients did not work as patients acquired resistance, and the de novo mechanism of resistance identified KRAS mutation that was initially not detected in the excised tumor tissues. However, the mutant gene responsible for the resistance was present in the circulating tumor DNA (ctDNA) isolated from the peripheral blood samples [7]. Therefore, it looks plausible to infer tumor biomarkers in the systemic circulation and other liquid biopsies that refer to circulating biomarkers in the form of DNA, RNA, proteins, and metabolites derived from tumors that can give a glimpse into cancer features [8–10]. If the diagnosis of neoplastic growth can be made upfront, the chance of mortality can go down; indeed, disease-free survival is possible in some cancer types, if not all [11].

The concept of liquid biopsy is based on tumor turnover; as the tumor grows, it releases cancerous cells into the blood circulation that can seed metastases. These cells or their biomolecules in the circulation, if detected/quantified, can lead to diagnosis [12, 13]. The specific and sensitive identification can reveal the type and anatomical location of the tumors. The FDA has approved diagnostic kits that utilize CTCs, and ctDNAs in patients where tissue biopsy excision is clinically not possible, like Guardant360 (https://www.accessdata.fda.gov/cdrh_ docs/pdf20/P200010A.pdf) and Foundation One Liquid CDx (http://www.accessdata.fda. gov/cdrh_docs/pdf19/P190032A.pdf). Although RNA is not considered as a viable analyte due to its unimpressive stability in the blood, but microRNAs (miRNAs), long non-coding RNAs (lncRNA), and circular RNAs (cirRNA) in circulation have shown promise in the context of liquid biopsies [14]. Lately, nanotechnology has also contributed to tumor diagnosis as it can also exploits the tumor exosomes, supposedly enriched in DNA, RNA, and proteins [15]. Moreover, with the advent of more sophisticated mass spectrometry, tracking the altered cancer metabolism that is usually cell-specific, has given rise to neo-metabolite, that can be utilized for designing diagnostic and therapeutic strategies [16].

Lung, liver, brain, and bone are the major metastatic sites and encompass specific characteristics leading to organ-specific tropism of cancer cells [17]. As in the case of BC, relapse occurs even after years of primary tumor removal, suggesting that these cells could have acquired specialized features to gain access to specific organs [18]. BrM is the most lethal and unique among various metastases. It requires cells that can breach the BBB, an anatomical challenge that also threatens the success of systemic therapies for brain cancer and other intracranial diseases. In BrM, it was surprising that the mutations that were commonly associated among multiple metastatic samples were often absent in the primary tumors from where BrM originated [19, 20]. Most of our knowledge is derived from in-vitro studies where enriched cells were obtained by the trans-well endothelial assay or in-vivo by injecting them through intra-cardiac/−carotid routes and isolating these cells from the brain. These enriched cells, thereby, show the capability to seed, colonize, and subsequently grow in distinct brain microenvironment [21–25]. Our group has shown a model where orthotopically transplanted BC cells to mammary fat pad led to induction of primary breast tumor that also metastasized to the brain [26]. This way, various hypotheses can be tested with a rationale to fetch out molecules and the pathways driven by these effector molecules that can be causal factors for BrM. Taking cues from artificial intelligence (AI), an Organ-On-a Chip 3D model was conceived, where tumor cells traversing the BBB can be studied. This can help design and interpret studies to understand the intrinsic phenotypic variations that could be a potential tool in BrM [27].

The compelling evidence that the ctDNA showed similar mutations present in lung cancer (LC) tissue samples led the FDA to approve the *EGFR* mutation-based liquid biopsy test to predict if tyrosine kinase inhibitor therapy would be responsive [28]. Similarly, immune cells infiltration in the brain microenvironment and their characterization through a non-invasive method like CSF analysis can help determine the response to various therapeutic interjections like the feasibility of immune checkpoint inhibitors (ICI) in BrM patients [29, 30]. Presumptively, the outcomes of blood-based biomarkers can be enhanced with available screening methods for multiple cancer lesions in various organs. The outcome has come out to be exciting in some studies, so much that only 1% showed false-positive blood tests [31].

The recent advances in understanding and non-invasiveness of liquid biopsies put forward its relevance in cancer diagnosis. Researchers or clinicians have employed its uses not only in clinical settings but also in deciphering the mechanism associated with metastasis [32]. Therefore, it is important to discuss our current understanding of the plethora of diagnoses dependent on liquid biopsies and major advances made in the realms of BrM research during the last decade. In this review, we acknowledge the potential biomarkers associated with metastasis, specifically those that form secondary tumors in the central nervous system (CNS). We have discussed CTCs, ctDNA, micro-RNAs, circular RNAs, lipids, proteins, metabolites, and exosomes that can be detected in body fluids **(**Table 1**)**. The compiled information in this review can help to narrow down on potential biomarkers for early diagnosis, prognosis and tracking the treatment response in BrM patients.

**Table 1:** Liquid biopsies based molecular determinants of BrM in various cancers

S. No.	Sample	Cancer Type	Molecular Determinants	Readout	Reference
1	CTCs/Blood/ (*N =* 10)	BC	EpCAM (+ve) /Notch Signaling	Upregulation in Notch Signaling	[33]
2	CTCs/ctDNA/ Blood/(*N =* 10)	LC	NRF2	Mutations in NRF2 (R34G)	[34]
3	CTCs/Blood/(*N =* 4)	BC	Semaphorin 4D/GPX1/MYC	High expression of SEMA4D, GPX1 and MYC gene amplification	[35]
4	CTCs/Blood/(*N =* 38)	BC	HER2+/EGFR+/HPSE+/NOTCH1+	Brain metastasis selected markers	[36]
5	CTCs/Pleural effusion/(*N =* 3)	BC	ST6GALNAC5	The high expression of ST6GALNAC5 facilitates co-option and passage through BBB	[37, 38]
6	CTCs/CSF/(*N =* 13)	BC	Syndecan-1 and MUC-1	Overexpression of syndecan-1 and MUC-1 in Leptomeningeal metastasis	[39]
7	ctDNA/Serum/(*N =* 311)	NSCLC	EGFR	BrM associated with the EGFR mutation and in concordance with EGFR status in tissues	[40]
8	ctDNA/Blood/(*N =* 28)	Metastatic Brain tumor	ALK, MDM2	BrM associated with the ALK and MDM2 mutation	[41]
9	ctDNA/Blood/(*N =* 70)	BC	miR124-2; miR3193; CCDC8	BrM associated with miR124-2, CCDC8 hypermethylation, and miR3193 hypomethylation	[42]
10	ctDNA/Blood/(*N =* 205)	LC	TGF-ß1	rs1982073 mutant	[43]
11	ctDNA/CSF/(*N =* 21;12)	LC	EGFR	> 50% patients have EGFR mutations	[44, 45]
12	ctDNA/Blood/(*N =* 92)	Melanoma	BRAF/NRAS	BRAF (V600E/K/D); NRAS (Q61R/L)	[46]
13	Protein/Serum/(*N =* 379)	LC	Cathepsin F/ Fibulin-1	Cathepsin upregulates in LC patients’ serum	[47]
14	Protein/Serum/(*N =* 29)	Brain Metastatic	C-Reactive Protein	C-reactive protein was upregulated in brain metastatic patients’ blood as compared to glioblastoma	[48]
15	Protein/Serum/(*N =* 120)	LC	IL6	Elevated IL6 in serum associated with BrM	[49]
16	Protein/Serum/(*N =* 30)	LC	S100ß	Serum has significantly high S100ß	[50]
17	Protein/Serum/(*N =* 68)	LC	Myelin	Myelin is high in serum	[51]
18	Protein/CSF/(*N =* 45)	Leukemia	sVEGFR-1,2	Elevated serum levels of sVEGFR-2	[52]
19	Protein/Serum/(*N =* 147)	BC	Glial fibrillary acidic protein	Elevated serum levels of GFAP	[53]
20	Protein/Serum/(*N =* 244)	BC	Tau	Elevated serum levels of Tau	[54]
21	Protein/Serum/(*N =* 68)	LC	Neurofilament Light Chain	Elevated serum levels of NFL	[55]
22	Protein/Serum/(*N =* 103)	BC	CXCL13; CX3CL1	CXCL13 and CX3CL1 enhances BBB permeability	[56]
23	Exosome/(*N =* 75)	LC	Integrinβ3	BrM occurs in patients with high EV ITGβ3 levels	[57]
24	Exosome/(*N =* 104)	BC	CEMIP	Tumor derived exosomes enriched in CEMIP protein promoted BrM	[58]
25	Exosome/(*N =* 6)	LC	miR-550a35p	miR-550a-3-5p controls the BrM	[59]
26	Exosome/(*N =* 38)	BC	miR-105	Cancer secreted miR-105 destroys BBB	[60]
27	Exosome/(*N =* 65)	LC	miR-335-5p/miR-34b-3p	miR-335-5p & miR-34b-3p are unique in BrM	[61]
28	Exosome/(*N =* 56)	BC	miR-181c/miR-503/miR-105	Enriches exosomes promotes BrM	[62, 63]
29	Serum/CSF/(*N =* 118)	BC	miR-200a, miR-200b, miR-200c, miR-141	Upregulated in BrM	[64]
30	Serum/(*N =* 24)	SCLC	LncRNA XR_429159.1	Downregulated in BrM	[65]

### Circulating tumor cells

Circulating tumors cells (CTCs) or disseminated cancer cells are continuously shed from the tumor, survive in the bloodstream and have shown potential for seeding secondary tumors at new metastatic locations [66]. CTCs can house at metastatic sites and go dormant, which can eventually come out of dormancy triggered by various mechanisms that are still poorly understood [67]. It has been postulated that the CTCs usually arise early during the primary tumor formation, as mutations found in metastatic lesions are usually different compared to the primary lesion, and then both primary and metastatic lesions evolve separately [68–71]. The kinetics of CTCs is different in various cancers, may be poised by their propensity to metastasize, that is usually several thousand of cells shed from a gram of tissue in the circulation, and this can go higher up to 700,000 cells per day as in the case of small cell lung cancer (SCLC). The half-life of CTCs in circulation is around several minutes [12]. Therefore, thousands of these CTCs remain in circulation and can seed metastasis in a spatially differentiated manner. Thus, it looks promising to target CTCs that have the propensity to drive towards a particular organ and characterize these cells.

In addition to being a tool for diagnosis and prognosis, CTCs can be instrumental in determining the molecular landscape of cancer where tumor acquisition is not clinically possible [72]. Studies revealed how BCs with a particular transcriptomic profile were often seen to metastasize to the brain. In those patients, Notch signaling and genes that control immune evasion like *CXCL8*, *CXCR4, *﻿and* CD86 * were upregulated [33]. In another study, SCLC patients were stratified into chemosensitive/refractory based on copy number variation (CNV) in the CTCs isolated from blood [73]. Now, an early diagnosis can be envisioned if CTCs, isolated from a patient’s blood or other body fluids, could be questioned for BrM based on their genetic profile. In BrM, besides blood or any other body fluids, CSF can be an ideal matrix as it has exclusive CTCs not available in the systemic circulation or in the extracranial region [74, 75].

**Fig. 1:**
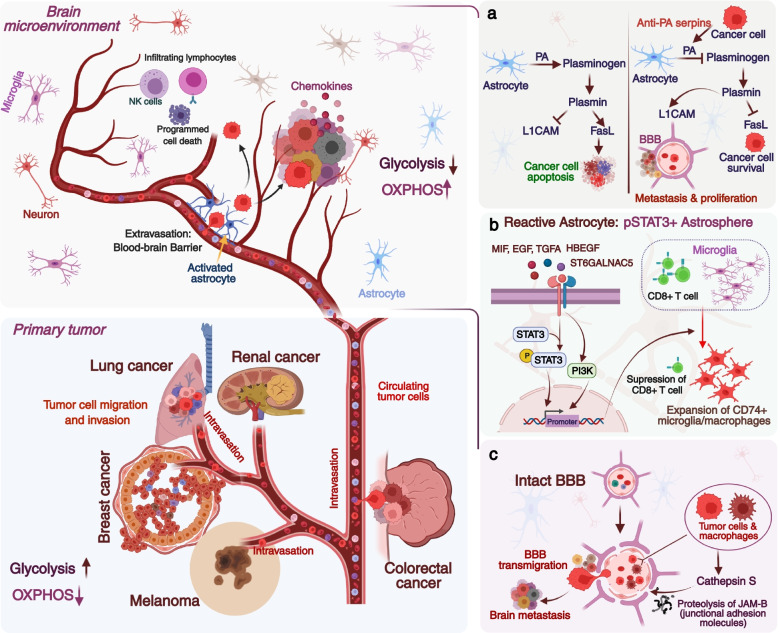
Brain metastases occur when cancer cells migrate from their primary site commonly lung, breast, colon, kidney, and melanoma to the brain. Circulating tumor cells (CTCs) or disseminated cancer cells are continuously shed from the tumor that survives in the bloodstream and can seed secondary tumors. The CTCs can house at metastatic sites and go dormant, which can eventually come out of the dormancy triggered by various mechanisms. **(A)** Brain stroma has plasmin that converts the astrocytic FasL into paracrine death signal for the metastatic cancer cells and inhibits L1CAM, needed for vascular co-option and metastatic outgrowth. In brain metastasis, anti-plasminogen activator (PA) serpins inhibit the plasmin (via inhibiting plasminogen activator) that guards the cells against FasL attack and activates the L1CAM that helps in vascular co-option of the brain metastatic cells. **(B)** STAT3 was found to label a subpopulation of astrocytes that were reactive and required for BrM. Brain metastatic cells had upregulated cytokines like MIF, TGF-α, and EGF that induced the STAT3 activation via phosphorylation leading to astrospheres formation that was capable of suppressing CD8^+^ T-cells. Reactive astrocytes also induce MIF to activate the MIF-CD74 axis to promote the outgrowth in BrM. **(C)** Cathepsin S proteolytically cleaves the junctional adhesion molecules, JAM-B in blood-brain barrier and helps in the transmigration of brain metastatic cells. Cathepsin S is elevated in primary tumors as well as in the macrophages of the stroma in TME

In the quest to figure out what pattern is observed in CTCs metastasizing to the brain, the CTCs derived from patients were injected into mice, and the metastasis was studied. This revealed intrinsic properties of CTCs like the new role of SEMA4D in cooperation with MYC and GPX1 as a mediator of extravasation and subsequent BBB transmigration [35]. Similarly, Cathepsin S (CTSS) is implicated in proteolytically cleaving JAM-B’s junctional adhesion molecules. Studies have indicated elevated CTSS in primary tumors and the macrophages of the stroma in the brain metastatic tumor microenvironment (TME). On depletion in both tissue and stroma, inhibited metastasis **(**Fig. 1**)**. However, no significant changes in tumorigenesis were reported [76, 77]. Cathepsin expression in serum, as seen in NSCLC and the microenvironment through the CSF, could help assess its role in non-invasive imaging and diagnosis [78, 79]. Lung cancer-derived BrM led to the release of CTCs in blood with mutations in KEAP1-NRF2-ARE pathway genes [34]. The cytoprotective role of the genes in response to stress points to their pro-survival in circulation, which otherwise is highly inefficient. The study suggests that the mutations in the Keap1-Nrf2-ARE pathway led to higher expression of antioxidant and detoxification enzymes that eventually could help cells survive in blood circulation and metastasize to other distant organs. We have further discussed the mutation in the ctDNA section. It was also revealed that CTCs have tropism, and only certain CTCs exclusively metastasized either to the brain, bone, or liver. Now, this is interesting as not only diagnosis is possible, but therapeutics can be tailormade by exploiting the disruption of the interaction between SEMA4D and Plexin-B1 and targeting CTSS, which can eventually be designed to stop cells from breaching the BBB (43,44).

In a BC study, patients with BrM had certain CTCs that were EpCAM negative; however, the same CTCs were EGFR, NOTCH1, and HPSE-positive [36]. This study was unique as it target EpCAM negative cells that cannot be captured through the FDA cleared CTC testing kit CellSearch® platform as it targets only the EpCAM positive cells, thereby increasing the range for CTCs implicated in BrM. Cancer cells devoid of the signature were non-metastatic, showing the critical importance of HPSE in metastasis [36]. In BC, HER2+ cells are the major cell types that metastasize to the brain specifically; it is interesting to note that in triple-negative breast cancer (TNBC) that metastasize to the brain also has HER2+ CTCs in the blood [80–83]. HER2+ belongs to the EGFR group of transmembrane receptors involved in tyrosine signaling and acts as a proto-oncogene. It is still being investigated the plausible explanation for BrM in such subtypes [84].

Slow cycle cancer-initiating cells, in blood circulation were found to be enriched for the stemness features, and most of them are EpCAM positive along with CD44 and CD24, and these are competent enough to seed secondary metastasis [85–87]. Stemness markers Oct4/Sox2, NOTCH, and WNT positive cells were enriched in the slow cycle subpopulation that eventually developed BrM. The association of the stem cell markers with BrM was significant as less than 1% of total cancer cells expressed these markers and were designated as the slow cycle cells [87]. The CSCs have been hypothesized to metastasize, and the existence of stemness marker-specific positive cells in the circulation could be utilized for diagnosing a probable BrM [88, 89].

Epithelial-mesenchymal transition (EMT) is induced by TWIST1 upregulation in LC and BC BrM samples. In these samples, lower E-Cadherin (involved in cell-cell adhesion) expression was observed, showcasing that TWIST1 could affect E-Cadherin downregulation [90]. Other EMT genes like *SOX2, EGFR, *and* c-MET* upregulation have been studied in CTCs of glioma patients but specifically, if these can be employed to diagnose BrM still needs to be ascertained [89].

In patients’ blood samples, a subset of EpCAM-negative CTCs having expression for urokinase plasminogen activator receptor (uPAR) and integrin β1 (int β1) were also found to be contributing to BrM [91]. The study shows that if CTCs are isolated with high uPAR or integrin-β1, it would probably hint us towards BrM, and these CTCs can be harvested from either blood or CSF. Although finding the CTCs in CSF will be more conclusive. The CTCs from the pleural effusion of BC patients were enriched for BrM and revealed that these CTCs also express *COX2* (PTGS2) and growth factor receptor (GFR) ligand HBEGF that help them in the extravasation whereas, ST6GALNAC5 promotes breaching of BBB [37]. In BC BrM, N-myc downregulated gene family (*NDRG1*) involved with DNA repair has shown to be pivotal in driving cancer cells to the brain [92, 93]. Knockout of *NDRG1* halted the BrM, which was otherwise close to 100% in cells with high positivity for NDRG1 [94]. Thus, CTCs with high NDRG1 could make the patient susceptible to BrM; however, further studies are warranted to validate their diagnostic capabilities.

Interestingly, EpCAM negative CTCs also expressed marker genes like *EGFR, ALDH*, urokinase plasminogen activator receptor, mucin1, syndecan-1, and caveolin1, that could be associated with a high propensity for BrM and exploited as a signature for BrM [36, 39]. However, the presence of EpCAM does have the potential in demarking the distant metastasis, though the frequency of CTCs obtained was less in BrM than in other organ metastases [95]. Some small cohort studies have revealed that patients with oligometastases have a better prognosis compared to patients having multiple metastases; however, the oligometastatic patients with high CTCs have a poor prognosis, and thus CTCs can help to stratify patients eligible for specific/targeted therapeutic strategies [95–97].

Consistent data has shown that T-Cell infiltration in brain metastatic lesions and isolation of T-cells from CSF are of the same clonotypes, indicating an interaction among them, which can be subsequently exploited for non-invasively predicting response to immune checkpoint inhibitors (ICI) and development of prognostic biomarkers [98]. Like cytotoxic lymphocytes isolated from CSF were characteristically similar to the tumor lesions, allowing to monitor inflammation on treatment with ICI [99].

### Circulating tumor DNA

Cell-free or circulating tumor DNA (ctDNA) in cancer patients’ blood was first described in 1948, and later studies indicated its level to be high, specifically in cancer patients with genomic alterations in tumor suppressor gene, oncogenes, microsatellite instability/epigenetic alterations derived from a tumor. However, information regarding their release mechanism and characteristics is still limited [100–105]. Possible routes of ctDNA in blood could be from apoptotic cells or macrophages engulfing the necrotic cells [106]. Unlike CTCs, ctDNA isolation does not require enrichment for specific rare cell type populations and is usually preferred for genotyping or studying drug responses [107]. ctDNA is not limited to cancer but also to other pathologies like autoimmune disease, cardiac dysfunction, inflammation, and even in pregnancy, though it gets elevated in cancer with the fragments of mutant DNA being detected in blood [100].

During metastasis, collateral tissue damage is seen in patient serum through elevated cfDNA having specific methylation patterns and could be used to correlate with distant organ metastasis [108–111]. A recent study found elevated hepatocyte-derived cfDNA and markers for liver damage, aspartate transaminase (AST), and alanine transaminase (ALT) were significantly correlated in liver metastasis, which was absent in healthy controls [112]. In patients with BrM, cfDNA from neurons, oligodendrocytes, or astrocytes having distinct methylation patterns were significantly upregulated in the serum samples as compared to other cancer patients or patients with metastasis to extra cranial regions [112].

The level of ctDNA is not merely dictated by tumor volume, but it also depends on the genotype of the ctDNA; for example, in the case of NSCLC, it depends on mutation in *EGFR* or *TP53* gene [113]. Suppose ctDNAs with specific mutations can be detected in the blood associated with malignancy. In that case, it can aid in screening for relapse and can be tracked for ctDNA clearance as the presence of detectable ctDNA is associated with poor survival, and undetectable ctDNA has higher survival [114]. A study on Korean NSCLC populations (*n =* 311) showed a significant association of EGFR mutation status with the BrM, the status of EGFR was the same in 71% of tissue and serum samples [40]. In some studies, clonal evolution was tracked by genotyping the ctDNA in blood. It revealed how *KRAS* mutation in a small subclone of cells evolved from tumors predominantly having WT-*KRAS*, thereby generating resistance to EGFR antibodies. Interestingly, on the withdrawal of the antibody treatment, the ctDNA with *KRAS* mutation declined in the blood, further strengthening the importance of tracking clonal evolution post clinical interventions [115, 116]. It has been suggested that ctDNA after surgery could be a primary prognostic marker; and also post-chemotherapy ctDNA analysis can stratify patients. The high-risk patients prime unique opportunities to explore aggressive therapeutic approaches. Treatment of patients considering the ctDNA level but no radiological evidence of disease after adjuvant chemotherapy could, in theory, even eradicate the minimal residual disease and increase the chances of cure [117]. ctDNA can guide the understanding of the progressions in the metastatic cascade from monoclonal or polyclonal seeding [118, 119].

In LC BrM, TGFβ1 is known to influence metastasis, there is one mutation in *TGFβ1* having a variant corresponding to rs1982073. This mutation is associated with poor survival, and metastasis to the brain is quite evident in NSCLC patients’ samples (*n =* 205) having received radiation as a part of the treatment regimen [43]. EGFR pathway is also critically involved, as prospective studies revealed, in how a mutation in the *EGFR* gene is correlated with BrM and, in some instances, with the number of metastases [120, 121]. Detection of ctDNA in CSF and blood revealed mutations in the *EGFR* gene and their concurrence with BrM. The frequency of ctDNA in CSF was higher up to 90% cases and around 60% cases in blood samples in the patients [44, 45]. *ALK* translocation and amplification have been reported in BrM with RET gene fusion; however, their clinical implication is still in its infancy [122, 123]. Mutations in *KEAP1*, *NRF2,* and *P300* genes are also associated with LC BrM, which could help cells to survive in circulation [34]. Usually, the interaction between KEAP1 and NRF2 is lost on mutations in either of the genes. However, in CTCs, NRF2 neh2 domain was found to have R34G, E79Q and E82G mutation, that eventually led the NRF2 to translocate to nucleus that otherwise, was located in the cytoplasm and changed the transcriptional profile of the CTCs [34, 124].

PIK3CA activation was quite evident as it helps in fatty acid generation; thereby, activating mutations in *PIK3CA* are the primary lookout in the ctDNA of HER2+ positive cells [125]. This looks relevant when we see HER2+ BC metastasizes to the brain, and the TNBC showing BrM can have HER2+ cells in the circulation [80]. Genetic alteration in ALK, MDM2, ATM, BRCA1, FGFR1 and KRAS were associated with BrM and the ctDNA from peripheral blood of the patients were persistent with the same mutations as seen in fresh tissue samples [41]. The deletion of PTEN, a negative regulator of the PI3K-AKT pathway, contributes to the upregulation of the PI3K-AKT pathway and activates NF-kB signaling [126]. CtDNA has also been exploited to assess the clinical implications longitudinally. BrM from LC involved the use of 100 TRACERx (TRAcking non-small cell lung Cancer Evolution through therapy (Rx)), tracking the evolution of ctDNA profile with longitudinal therapy, paving the way for ctDNA driven therapeutic modalities [18]. Besides all these genes, SERPINI1 was also frequently mutated in BrM patients. This might help metastatic cancer cells to adapt in the circulation and eventually extravasate to form metastases [127]. Epigenetic changes are usually seen in metastatic samples, and in some studies, they have tried to use a serum to read the difference in the epigenetics of primary tumor and metastatic cancer cases [128]. In one such study, methylation pattern was studied for *miR124-2*, *CTD-2028 M8*, *CCDC8,* and *miR3193* in primary and ctDNA. There was 100% concordance with the tissue status in *miR124-2* and *CTD-2028 M8*. However, *CCDC8* showed 80%, and *miR3193* had a 50% similar status [42]. Out of these genes *miR124-2, CCDC8* were found to be hypermethylated, whereas *miR3193*was hypomethylated. Lung cancer genome-wide DNA demethylation was studied, and methylation patterns in tissue and blood samples were concordant. The patients’ sample reflected poor global methylation in BrM [129].

In melanoma BrM, BRAF or NRAS mutations are more prominent [130]. V600E/K is among the mutation in BRAF, and NRAS has a mutation at Q61/G12/G13 associated with BrM [46, 131–135]. These mutations were found in ctDNA from the blood of metastatic melanoma patients [136]. A subsequent mutation in STK11/LKB1 makes it more invasive towards the brain. They are involved with activating phosphorylation STAT3/5 and the FAK pathway [137]. All these studies were done on solid tumor, however, during a clinical trial, it was found that 84% cfDNA, BRAF status and tumor mutation status was in agreement, and this could be a viable option as a prognostic marker [46].

### Proteins and metabolites

Cytokines and chemokines have a prominent role in metastasis, in fact, several studies have shown how the seed and soil concept is recapitulated in the context of the chemokine’s functioning [138, 139]. One study revealed how LC cells when treated with TGFβ1 protein and were injected in mice, the chances of BrM were 3-folds higher than the cells that did not get any pretreatment with TGF-β [140]. Reduction in secreted CTSS protein, as discussed earlier in circulating tumor cells, led to diminished BrM, which can be evaluated as a biomarker [76, 141]. With PTEN loss in brain metastatic tumor cells, CCL2 secretion is enhanced, leading to the recruitment of the myeloid cells that drive proliferation [126]. However, not only chemokines or other small immune factors, but many proteins, especially like EGFR, HSPG2, FASN, FN-1 or PYGB can be detected in blood and are also implicated in BrM [142]. JAK2/STAT3 signaling is also shown to be involved in BrM, and IL6 could be initiating this axis, and it has been found to be upregulated in serum samples of NSCLC patients, significantly correlating with BrM [49]. Annexin A1 was also found upregulated in the sera of the SCLC patients, and annexin A1 is involved in trans-endothelial migration as knockdown of it led to diminished trans-endothelial migration in-vitro and prevented brain metastasis in mice [143]. Similarly, S100B was elevated in the serum of NSCLC patients, and later it was found that S100B was implicative in BrM [50, 144]. The same results were seen in SCLC, which also showed elevated expression of S100B in the sera of the patients with BrM [145]. Later it was revealed that S100B autoantibodies could also be used to identify BrM patients [146]. In another study where HER2 and S100B were analyzed in serum, only HER2 levels in serum were correlated with the BrM [147].

In LC, the expression of myelin basic proteins was upregulated in the sera of patients with BrM [51]. This upregulation could be due to the breaching of BBB that could have led to the build-up of myelin in the serum. Another important study exploited the prognostic capability of drug resistance protein that later revealed the increased expression of multidrug resistance protein (MRP) and in LC metastasizing to the brain [148]. MRP is thought to be involved with the prevention of the influx and promotes efflux at the BBB, and therefore chemotherapeutic drugs are ineffective [149].

The tumor microenvironment within BrM was enriched for VEGF-A, TIMP-1, extracellular matrix proteins (ECMs) and Lipocalin-2 molecules that are also implicative in immune suppression [150–155]. Soluble VEGFR-1 was also found to be increased in the BrM patients’ serum and CSF [52, 156, 157]. Thereby, when tyrosine kinase inhibitor targeting endothelial VEGF was used, it inhibited angiogenesis with a concurrent decrease in BrM. A recent study assessed a panel of proteins in serum and found glial fibrillary acidic protein associated with the potential of BC to metastasize to brain; however, this still needs to be assessed in different or large cohorts of patients [53]. In a patient study, C-reactive protein levels in the blood distinguished the brain metastatic patients from glioblastoma, that included 29 patients; however, primary tumor origin was unknown, but this could be a good diagnostic marker if it stood thorough validations [48]. Similarly, neuron specific protein neurofilament light chain (NfL) release due to neuron degradation was found to be high in serum samples of patients with BrM, and also high NfL patients had poor survival [55]. Cytokine CXCL13 and CX3CL1 were high in the serum of breast cancer patients, which could eventually led to enhanced permeability [56]. Tau protein, majorly located in CNS, involved in microtubule stabilization and polymerization, was evaluated for diagnosing BrM from BC, and it was revealed that Tau level in serum could independently predict BrM [54]. Study on BC patients (*n* = 113) also found to express elevated levels of Angiopoietin-like 4 (ANGPTL4), which could help in trans endothelial passage of the BC cells via interacting with the integrin, cadherin, and claudin-5 [158]. 

A recent study showed that the astrocyte-derived laminin-211 sequestered YAP protein and held disseminated tumor cells quiescent at the astrocyte endfeet, and from these quiescent cells even a single cell on release could give rise to BrM [159]. Laminin-induced changes, if can be detected in the CSF or serum, can give a glimpse about a likely BrM as done in some neurological disorders like Alzheimer’s disease could help in understanding the diagnosis of BrM [160]. However, its use as a BrM diagnostic factor still needs to be investigated.

Renewed interest in cancer metabolism, the reprogramming in the metabolism of the tumor tissues with the advent of higher resolution mass spectrometry, led to the profiling of the metabolites through the targeted or untargeted metabolomics revealing neo-metabolites or oncometabolites elevated in body fluids that could be utilized for diagnostic and therapeutic implications [161–164]. Single-cell RNA sequencing data clearly showed that the primary BC cells were highly glycolytic, and the metastatic tumor cells exhibited more oxidative phosphorylation (OXPHOS) (Fig. 1). This could pave the way for further research into assessing these metabolites in the OXPHOS and can be put into research to look for diagnosis and therapeutics with prognostic assessments [165]. In fact, the first metabolite identified was 2-hydroxyglutarate (2HG) detected in glioma patients with a mutation in IDH 1 [164]. Diagnosis based on MR spectroscopy is based on the metabolites assessment and could noninvasively define a few metabolites in the brain [166, 167]. CSF-derived metabolites have consistently helped in discriminating different brain tumor types [168]. Brain metastatic cells going to the brain reprogram their metabolism to adapt the brain microenvironment, and this reprogramming lead to distinct metabolite features [168–172]. 

In a cohort, with 88 BC patients, out of which 33 had BrM, the serum metabolomics revealed some significant changes in various metabolites. In amino acids, alanine, valine, proline, glycine, serine, threonine, phenylalanine, and arginine were upregulated. In sugar, fructose and similarly, lipid sphingosine, besides fumarate, lactate, and pyroglutamate all were found to be higher in the serum of the BrM patients. These metabolites were responsible for the amino acyl tRNA biosynthesis and nitrogen metabolism and these were upregulated in brain metastatic patients [173]. However, more exhaustive, and multi-center data will help reach a consensus. Another interesting study indicated a correlation between metabolic fingerprints to BrM in animal models [174]. Mice were injected with B16F10, MDA-MB-231 BR, and 4 T1-GFP cells intracerebrally or intracardially, and a predictive model was achieved based on the urine metabolites that indicated both sensitivity and specificity. The metabolites detected by NMR spectroscopy in the urine showed differential expression of allantoin, citrate, trimethylamine, trimethylamine-N-oxide, 2-oxoglutarate, creatinine, taurine, and creatine with phosphocreatine [174]. These metabolites were time-dependent and able to diagnose early tumor progression. However, allantoin being present in all models with BrM could not be a marker for diagnosis as human metabolism ceased before allantoin at uric acid. In SCLC patients, high serum lactate dehydrogenase (that converts pyruvate to lactate) was found to be associated with BrM [175]. Though LDH has been shown to be associated with poor survival in LC but its relevance in respect to BrM and elevated levels in serum make it a favorable candidate for the diagnostic marker. In BC also, LDH levels have shown the potential to predict BrM [176].

The field of metabolomics has grown exponentially in the past decade, with new molecules with respect to cancer metabolism, being detected and added to the repertoire of the oncometabolite. If any differentially expressed oncometabolite can be detected and is quantifiable, it could resolve the qui vive for a diagnostic marker for BrM.

### Extracellular vesicles

**Fig. 2:**
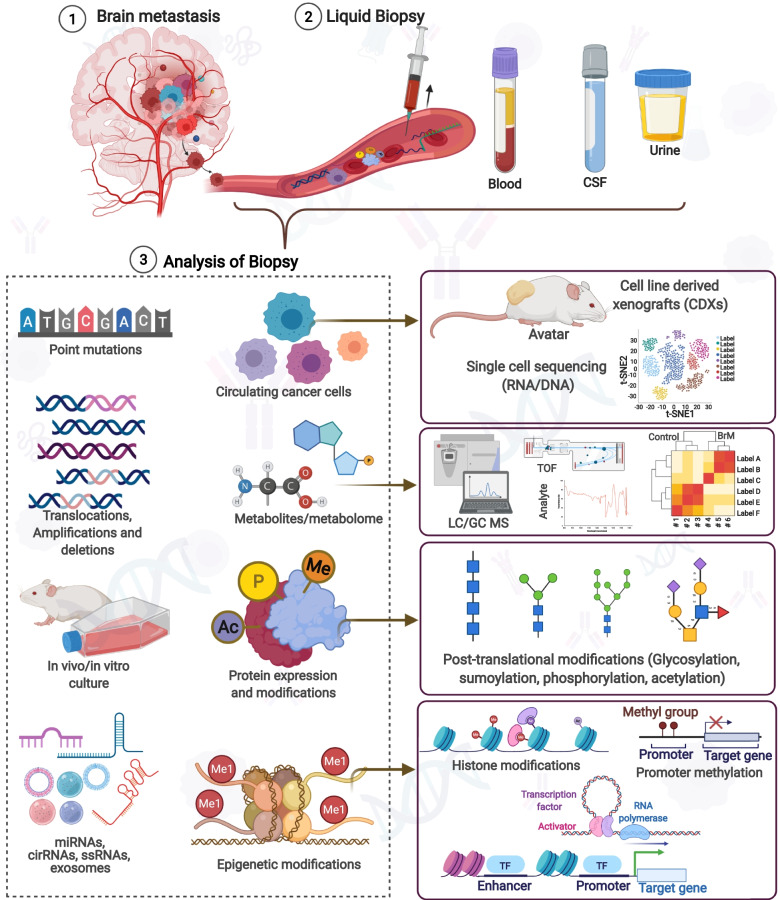
Cells and various factors are shed by the tumor into the circulation that can be harnessed for liquid biopsy. Blood, CSF, and urine are the analytes that can be targeted. Usually, CTCs can give a glimpse of various abnormalities associated with DNA, RNA, proteins. ctDNA can be targeted to infer mutations, translocation, deletion, or amplification. Exosomes are enriched with metabolites and proteins besides various non-coding RNAs which can be present in cell-free form too. High throughput technologies like NGS, single-cell sequencing, proteomics, epigenetics, and metabolomics can unfurl these target biomolecules, which can be implicative in BrM

Extra cellular vesicles (EVs), also known as microvesicles, microparticles, ectosomes, exosomes, oncosomes, and apoptotic bodies are the lipid membranous structures of diameter 30-150 nm released by the cells in the normal physiology of cells usually for intercellular communication, immunity or in different pathological conditions like inflammation, neurodegeneration or in cancer [177–181]. In cancer, billions of EVs increases and selectively upregulates the contents that contain DNA, RNA, microRNA, long noncoding RNA, circular RNA, lipids, proteins, and various metabolites, which are pivotal not only in cancer progression but in metastasis that could be exploited at diagnostic and therapeutic axes [177–182]. The disseminated cancer cells need to adapt, survive in the different microenvironments and proliferate. EVs are critically involved at this junction, with their repertoires modulating the changes after the interaction with the tumor cells and EVs, which can breach the physiological barriers [126, 178, 183, 184]. The status of these biomolecules in EVs exposed to patient body fluids like blood, CSF, saliva, pleural effusion, and ascites, as secretory biomolecules can dictate the development of non-invasive biomarkers for BrM [185] **(**Fig. 2**)**.

EVs are critically involved in modulating the TME in a spatio-temporal manner, influencing tumor growth and enhancing distant metastasis [186, 187]. It has been shown that EVs could be involved in the preparation of premetastatic niche, in fact, EVs isolated from cancer cell lines with different propensities to metastasize to the distinct region were found to be populous at the future metastatic sites, and specific cells interacted with the EV for their subsequent uptake [188]. The EVs express specific surface proteins, which could assist in origin prediction, that further help in predicting their favored metastatic site [189]. These EVs could even help prepare the pre-metastatic niche for the cancer cells that were earlier incapable of homing [187–189]. Due to heterogeneity in EV size, capacity, and content, isolation of EVs has become a cause of primary concern. Various protocols have been developed to isolate EVs from plasma that can be implicated in diagnosis, therapeutics, and monitoring the treatment regimen [190–193]. EVs having miR-193a, miR-25-3p, miR-141-3p were found to be involved in liver metastasis, this open avenue that tumor-derived exosomes could be assessed for their possible inherent role in BrM [194–196]. Melanoma-derived exosomes disrupted the integrity of the BBB and induced microglia activation [197]. Besides diagnosis or prognosis, the treatment regimen poses several challenges, including drug efficacy or resistance, which can be addressed following exosomes isolation and characterization. In BC cells, low miR-567 was associated with the trastuzumab-resistance [198]. Similarly, higher lncRNA-SNHG14 containing exosomes or lncRNA-CCAL encoding exosomes made colorectal cancer cells oxaliplatin resistance [199]. EVs can also predict immunotherapy response, as in the case of melanoma (usually have high propensity to metastasize to the brain), exosomes with PD-L1 expression were resistant to the anti-PD1 therapy [200].

### Proteins in EVs

Proteins are the most studied content of the EVs, which can be studied for proteomic profiling that conclusively segregates the tumor cells from the normal cells with 95% specificity [182, 189]. The multiple panels profiling was so defined and robust that they could predict the origin of the tumors. It is being postulated that the brain microenvironment requires conditioning for the cancer cells outgrowth, and this pre-conditioning can come from EVs elevated with proteins of interest, as shown by the treatment of brain tissues with EVs derived from brain metastatic cells that led to a fourfold increase in cell colonization and increased invasiveness [58]. Integrins interact with the extracellular matrix proteins in distant colonization, regulating cell survival, stemness, and metastatic potential [201, 202]. During metastasis to the lung and liver, the EVs are abundant with integrins, which could be investigated to ascertain the organ-specific metastasis; however, in the case of BrM, these exosomal integrins are fewer compared to other organ metastasis. Nevertheless, some recent studies with EVs of brain tropic cells showed upregulated integrin ITGβ3 [57, 188]. Integrin expression patterns can be inferred to ascertain the organotropism after the EV’s isolation.

Interestingly, CEMIP, associated with normal brain physiology as well as involved in cancer and inflammation, was found to be upregulated in EVs derived from brain metastatic cells. Hyaluronic acid depolymerization, cellular calcium, and WNT signaling modulation are some of the functions of CEMIP [58]. Another small molecule secreted by the leukemic EVs is IL15, which was internalized by the astrocytes that, alters their activation and increases the expression of VEGF-AA which ultimately compromised the integrity of BBB, and IL15 inhibition can decrease CNS metastasis [203]. In SCLC cells, there was a distinctive elevation in the S100A16 when cells were co-cultured with endothelial cells, and this effect was inhibited when exosome secretion was pharmacologically inhibited. Further, it was shown that the S100A16 is involved with the maintenance of the mitochondrial membrane potential, which actively ensures SCLC cells survival in the brain milieu [204]. This was striking as the secondary site was releasing factors that will help establish the cells of SCLC in the brain, which otherwise might die on entering the brain microenvironment. This opens the avenue when CSF can be investigated for the exosomes loaded with S100A16, making the brain susceptible to secondary tumor formations [204].

Fibronectin and cyclin D1 were among the highest expressing proteins in the exosomes of cancer cell lines expressing brain metastatic phenotype [205]. Fibronectin is involved with the adhesion, invasion, and metastasis of melanoma and BC cells [206]. Likewise, cyclin D1 overexpressed in BC and melanoma could be involved in TME modulation for the survival of metastatic cells in the brain microenvironment [207, 208]. In BC BrM exosomes enriched with annexin II was found and that helped in tPA-dependent angiogenesis [209]. The BrM was reduced by 4-fold when the annexin II depleted exosomes were used for priming that was regulating the macrophage activation via p38MAPK, NF-kB and STAT3 pathways.

### Non-coding RNA in EVs

MicroRNAs are relevant in cancer biology as their deregulation is being one of the hallmarks of cancer. Currently, microRNA-based therapies are conceived as a therapeutic option [210]. MicroRNA dysregulation and their transport in EVs or as circulating free microRNAs open a window for diagnostics, where microRNAs can decree for possible metastatic propensity to a particular organ [211, 212]. PTEN was found to be downregulated in brain metastatic BC cells relative to primary BC cells; however, it was restored when cancer cells left the brain microenvironment. This PTEN regulation was conceived through microRNAs secreted by the astrocytes [126, 213, 214]. This process shows the relevance of the microRNAs in BrM. Therefore, a differential expression of microRNAs in BrM from breast, lung, melanoma, and other primary cancer sites could help in early diagnosis **(**Table 1**) (**Fig. 2**)**.

XIST transcript, a long non-coding RNA, was downregulated in cancer cells that specifically metastasizes to the brain and not bone, promoting EMT and activation of c-MET. Loss of XIST induced the expression of miR-503 that decreases the M1-M2 polarization of the microglia leading to stunted T-cell proliferation [215]. In another study on primary BC and melanoma cell lines compared to brain metastatic cells, miR-210 was upregulated, and miR-19a and miR-29c were downregulated in brain metastatic cell lines [205]. miR-122 downregulates the pyruvate kinase, thereby suppressing glycolysis in non-tumor cells in the pre-metastatic niche, leading to higher nutrient availability for the metastatic cells [216, 217]. In BC patients with metastasis, a higher miR-122 expression was usually demonstrated, which has been implicated with increased glucose availability to the cancer cells for their survival in the brain parenchyma [218]. MiR-550a-3-5p was also found to be enriched in EVs from LC and were potentially overexpressed in EVs from LC brain metastatic patients [59]. In advanced breast cancer, serum shows elevated levels of miR-4428 and miR-4480 and this increase can even distinguish patients with brain metastasis [219].

In NSCLC, lncMMP-2 was highly expressed in the TGF-β mediated EVs that led the NSCLC to metastasize to the brain via breaching the BBB permeability [220, 221]. A study using CSF isolated from 65 patients and then microRNA identification using prediction analysis of microarray showed that miR-335-5p and miR-34b-3p were unique in NSCLC BrM specifically to the leptomeningeal metastasis samples [61]. In other studies, miR-423, miR-330-3p, miR-145 were found to have potential for diagnosis as they were dysregulated in LC BrM, but this profiling was not done regarding exosomes [222–226]. MiR-181c, miR-503, and miR-105 were some of the miR enriched in BCBM and involved in the deregulation of tight junctions like N-cadherin, ZO-1, which ultimately weakens the BBB [62, 63, 197]. Now, this is critical as this was quite an important revelation of the changes in the actin dynamics, which promoted the uptake of the cancer cells into the cranium. Among the tight junction studies, loss in claudin-5 has been implicated in BBB permeability, and subsequent BrM from lung and melanoma has also been reported [227, 228]. Therefore, it can be said that the presence of microRNA targeting the tight junctions will eventually allow any cancer cells in circulation with a high propensity to metastasize to the brain. In addition, as reviewed by Kanchan et.al. the miR-200 family was found to be upregulated in the serum and CSF of BC BrM [64]. Likewise, miR-132-3p, miR-199a-5p, miR150-5p, and miR-155-5p could also be exploited for diagnosis as well as for patient prognosis [229]. EVs interacting with astrocytes led to modulation of the brain matrix microenvironment for the subsequent metastasis, and these EVs were enriched with miR-301 [230]. In a very interesting experiment, melanoma cells were injected intracranially into mice and tumor growth was analyzed. It was found that as the tumor growth in the brain progressed the small EVs containing miRNA, in circulation was increased. These small EV were continuously enriched for human-specific miRNAs, which included miR-224-5p, miR-130a-3p and miR-21-5p with tumor progression [231].

Circular RNAs have been involved in tumorigenesis, and progression has also been implicated in BrM, like circBCBM1 (hsa_circ_0001944) identified in BC. It is being postulated that circBCBM1 acted as a sponge for miR-125a and miR-509 activity, resulting in heightened BRD4 and MMP-9 through the Sonic hedgehog pathway [232, 233]. Circular RNA hsa_circ_0052112 also appears to downregulate the miR-125a-5p in BC [234]. Interestingly in clinical samples of BC with BrM, circBCMB1 (hsa_circ_0001944) was upregulated in tumor tissues and plasma samples [232]. In the same study, other circRNAs such as hsa_circ_0001481, hsa_circ_0000646, hsa_circ_0001006 and hsa_circ_0000732 were also found to be upregulated and hsa_circ_0001910, hsa_circ_0008285, and hsa_circ_0000002 were downregulated in BC cell lines going to the brain. LncRNA GS1-600G8.5 is found in BrM EVs and is known to destroy the BBB by decreasing ZO-1, claudin-5, and N-cadherin [235]. Besides upregulated lncRNAs, low expression of LncRNA XR_429159.1 was a risk factor for SCLC BrM, regulating the neuroepithelial transforming gene 1 (NET1) pathway [65]. LncRNA associated with BrM was named Lnc-BM, enhancing the STAT3 phosphorylation through the JAK2-Oncostatin M and IL-6 axis. This led to ICAM1 and CCL2 expression mediating vascular co-option and macrophage recruitment [236].

## Conclusion & future perspective

With all its challenges, liquid biopsy will be the best option in future oncology care comprising diagnosis, prognosis, and keeping track of the minimal residual tumor and recurrence. It has the potential to significantly reduce cancer-related morbidity and mortality. On the other front, BrM, which has been lately the major problem associated with disease progression mainly in the case of BC, LC, melanoma or clear cell renal carcinoma, is devoid of any molecular biomarkers, which are currently in clinical practice. Therefore, understanding the molecular features in cancer cells- especially the disseminated ones that prime these cells to specifically metastasize to the brain, will help design better diagnostic strategies. This can be done by studying the CTCs, cell-free nucleotides ctDNA or non-coding RNAs in the form of microRNAs, circular RNAs, or long non-coding RNAs **(**Table 1**)**. Secretory proteins and metabolites have also shown their potential; however, there is a definite gap in our understanding about alien cells homing the brain.

Clinical trials are underway, and among them, one is studying the serum glutamate, aspartate, lactate, glutamate pyruvate transaminase, glutamic oxaloacetic transaminase, and lactate dehydrogenase levels in the BrM patients of LC, BC, and melanoma treated with stereotactic radiosurgery and patients with the same primary controlled tumors with neither brain nor extracranial metastases (ClinicalTrials.gov Identifier: NCT04785521) **(**Table 2**)**. Another clinical trial is now recruiting patients of BC (TNBC/HER2+), NSCLC, SCLC, melanoma, and other solid tumors who have been at high risk or close to a possible diagnosis of CNS metastases for the analysis of ctDNA from plasma and CSF samples (BrainStorm Program, ClinicalTrials.gov Identifier: NCT04109131) **(**Table 2**)**. More discoveries in this direction would enrich our portal that can be used to screen for potential BrM.

**Table 2:** Clinical trial on brain metastasis based on liquid biopsies

Clinical trial identifier	Liquid biopsy	Cancer type	Status	Outcomes/predicted outcomes
NCT04785521	Blood samples (serum)	BrM from melanoma, lung, and breast cancer treated with stereotactic radiosurgery (SRS)	Recruiting	a) Serum GOT1, GPT, LDH, glutamate, aspartate, and lactate determination and comparison in: -newly diagnosed BrM patients before SRS treatment. -melanoma, breast, and lung cancer patients without BrM. -newly diagnosed BrM and non-BrM patients. -patients carrying benign intracranial lesions (before and after SRS treatment). b) Studying correlation of serum markers with MRI changes following SRS treatment.
NCT03550391	Plasma and serum	Patients with BrM (all cancer types)	Recruiting	-Whether detectable somatic mutations from liquid biopsy could be able to predict overall survival of patients with BrM and development of new BrM. -Analysis of serum biomarkers such as C-reactive proteins and brain-derived neurotrophic factors to elucidate genomic changes or molecular mechanism of neurocognitive decline associated with BrM. -To compare overall survival in BrM patients who receive SRS treatment to patients who receive hippocampal-avoidant (HA-WBRT) radiotherapy.
NCT04109131 (BrainStorm)	Blood sample (for plasma & serum) CSF	TNBC/HER2+ BC, NSCLC, SCLC, and melanoma	Recruiting	-Epidemiology of CNS metastases and identification of risk factors for CNS metastases (including time to first CNS event and time to second or subsequent CNS events after first treatment). -Understand heterogeneity between the primary tumor and the CNS metastasis. -Identification of promising therapeutic targets for novel compounds. -Building clinico-pathological database for patients with newly diagnosed non-CNS metastatic solid tumors with high risk of developing CNS metastasis. -ctDNA analysis from CSF samples.
NCT03257735	Blood and CSF	NSCLC	Recruiting	Gene mutation status in CSF, blood, and tissues, and comparison of mutations after first session and during tumor progression to explore the role of liquid biopsy in the diagnosis and therapeutic advancement of NSCLC with BrM.
NCT02058953	Blood and CSF	Melanoma	Completed	To understand if melanoma CNS metastases are similar to primary melanoma, and development of biomarkers for the prediction of CNS metastases from primary melanoma.

Overexpression of neuroserpins and L1CAM could instigate BrM, which can be further integrated with GRIN2B and CTSS; as such, their expression makes cells competent enough to withstand the various attacks from the reactive brain stroma. CTCs isolated from blood and CSF can be evaluated if they harbor such anti-PA serpins, CTSS, and others to make metastatic cells competent in seeding metastatic lesions in the brain. The presence of such CTCs in blood will intimately augur a competent future brain infiltration. However, to exploit these features as an option for liquid biopsy, we need studies with large cohorts of patients with cancer prone to BrM.

DNA mutations can confer genes with the gain and loss of functions mutation. Some tumor suppressor genes become lethal on mutating and have been associated with highly aggressive cancers [237, 238]. *BRCA2, NOTCH, RB2, KEAP1, NRF2* were among the topmost mutated genes in metastatic BC cases. These mutations are found in the circulating tumor DNA and, besides diagnosis and therapeutics, it could also help in tracking the efficacy of therapy. These mutated circulating tumor DNA can also be found enclosed in EVs. Besides CTCs, EVs can also be a source of proteins, metabolites, cell-free RNA, or DNA, and non-coding RNAs **(**Table 1**,** Fig. 2**)**. The presence of proteins and metabolites can also be detected in serum; some of the proteins like carcinoembryonic antigen and carbohydrate antigen 19-9 are FDA approved to diagnose and monitor treatment effects. EVs with artificial intelligence hinted at detecting early-stage 1 and 2 pancreatic cancers. Liquid biopsies based on EVs are getting popular and could be a revolution in liquid biopsy-based cancer diagnostics [239].

Taking a cue from the vast literature on the molecules implicated in BrM, it could be possible to use these screenings for cancer progression. CTCs can be evaluated for the upregulated genes expression with copy number variations and the other biomolecules. EVs will encompass all the cell-free RNA, ctDNA, proteins, and metabolites. ctDNAs are the best analyte, giving instant access to mutations in the DNA. Thereby, this has led to the idea that if clinical trials on patients at a high risk of developing BrM are subjected to the screening of these potential molecular profiles, it can help stratify the patients into high or low-risk BrM groups. However, this comes across to look for the efficacy of the molecules in the early diagnosis. Except for relying on radiodiagnosis or the development of neurological symptoms, no biomarker is currently available for diagnosing BrM.

Liquid biopsies also have limitations that get even more aggravated when BrM has to be investigated due to provocative anatomical and physiological discrepancies. Primarily the seed of fatal metastases, CTCs are rare when the tumors are established but can be quite intuitive in the sense that they have cancer-associated abnormalities. When tumors are metastasizing and losing cells heavily in the circulation, then short half-life of DNA or circulatory cells, is another factor for delimiting the potential of liquid biopsies. However, isolating CTCs from the blood or other body fluids is challenging, implying the use of proliferation markers like Ki67, uPAR, or supplementing with antibody-mediated capture; again, all these strategies come with their limitations, specifically when non-epithelial cells are of interest [240]. In patients with BrM, it is even more challenging to isolate CTCs as they are less in numbers than extracranial metastasis [95]. In addition, liquid biopsies comparing CTCs, ctDNA, protein or metabolites either free or enclosed in EVs have potential when they can be clubbed with surgeries as post-surgical circulating DNA remnants pose a relapse threat as compared with patients with no circulating DNA features. However, the lack of sensitive technological advancement extrudes the confidence for taking the final call on the future perspective of the disease. Thus, it can only be used in conjunction with other factors assessment. The factors or cells secreted by the tumor and factors secreted by the secondary metastatic site for the successful homing. It is unknown if these factors can breach the BBB and can be spectacled in the circulation, so CSF examination is the best bet. Though drawing CSF is considered safe, but for longitudnal assesment doing lumbar punctures at regular intervals would be quite painful, and the patients usually have headaches after the procedure. In some extreme conditions, nerve damage or infections can make it worse.

Although significant advances in the understanding of BrM have been made, no absolute biomarker is available that is currently being used in clinical settings, and radiodiagnosis or PET-CT imaging is only good when the tumor has progressed to a particular size that can be imaged. Thus, in the future, it can be a possibility that radiodiagnosis and liquid biopsies can be combined to diagnose BrM at an earlier stage when the tumor burden is low. Also, it will help in separating the responders from non-responders. With technological advancement, more interest is currently being generated in liquid biopsies for BrM diagnosis and prognosis, and it could also prove to be effective in preventive oncology.

## Data Availability

Not applicable, all information in this review can be found in the reference list.

## Abbreviations

BrM: Brain metastasis
BBB: Blood brain barrier
CTC: Circulating tumor cells
CSF: Cerebrospinal fluid
ctDNA: Circulating tumor
lnRNA: Long non-coding
cirRNA: Circular RNAs
SRS: Stereotactic radiosurgery
AI: Artificial intelligence
FDA: Food and drug administration
EGFR: Epidermal growth factor receptor
ICI: Immune checkpoint inhibitors
PET: Positron emission tomography
CT: Computed tomography
CNS: Central nervous system
SCLC: Small cell lung cancer
NSCLC: Non-small cell lung cancer
CNV: Copy number variation
JAM-B: Junctional adhesion molecules
TME: Tumor microenvironment
EpCAM: Epithelial Cell Adhesion Molecule
HPSE: Heparanase
NMDAR: Neuronal glutamate receptor
TNBC: Triple negative breast cancer
VRB: Vinorelbine
TUBB3: Beta-3 tubulin
NF-кB: Nuclear Factor kappa B
MMP: Matrix metalloproteinases
MIF: Macrophage migration inhibitory factor
CD: Cluster of differentiation
EMT: Epithelial mesenchymal transition
GFR: Growth factor receptor
FASN: Fatty acid synthase
FABP: Fatty acid binding
VEGF: Vascular endothelial growth factor
TIMP: Tissue inhibitors of matrix metalloproteinases
DNAm: DNA methylation
EZH2: Enhancer of Zeste 2 Polycomb Repressive Complex 2
KRAS: Kirsten rat sarcoma viral
TOP2A: Topoisomerase 2 alpha
PTEN: Phosphatase and tensin homolog
EVs: Extracellular vesicles
